# CP12 fine-tunes the Calvin-Benson cycle and carbohydrate metabolism in cyanobacteria

**DOI:** 10.3389/fpls.2022.1028794

**Published:** 2022-10-11

**Authors:** Stefan Lucius, Marius Theune, Stéphanie Arrivault, Sarah Hildebrandt, Conrad W. Mullineaux, Kirstin Gutekunst, Martin Hagemann

**Affiliations:** ^1^ Department Plant Physiology, University Rostock, Rostock, Germany; ^2^ Molecular Plant Physiology, Bioenergetics in Photoautotrophs, University Kassel, Kassel, Germany; ^3^ Botanical Institute, University Kiel, Kiel, Germany; ^4^ Max Planck Institute of Molecular Plant Physiology, Emeritus Group System Regulation, Potsdam, Germany; ^5^ School of Biological and Behavioural Sciences, Queen Mary University of London, London, United Kingdom

**Keywords:** diurnal cycle, fluorescence tagging, photomixotrophy, glyceraldehyde 3-phosphate dehydrogenase, phosphoribulokinase, redox regulation, *Synechocystis* 6803

## Abstract

The regulatory protein CP12 can bind glyceraldehyde 3-phosphate dehydrogenase (GapDH) and phosphoribulokinase (PRK) in oxygenic phototrophs, thereby switching on and off the flux through the Calvin-Benson cycle (CBC) under light and dark conditions, respectively. However, it can be assumed that CP12 is also regulating CBC flux under further conditions associated with redox changes. To prove this hypothesis, the mutant Δ*cp12* of the model cyanobacterium *Synechocystis* sp. PCC 6803 was compared to wild type and different complementation strains. Fluorescence microscopy showed for the first time the *in vivo* kinetics of assembly and disassembly of the CP12-GapDH-PRK complex, which was absent in the mutant Δ*cp12*. Metabolome analysis revealed differences in the contents of ribulose 1,5-bisphosphate and dihydroxyacetone phosphate, the products of the CP12-regulated enzymes GapDH and PRK, between wild type and mutant Δ*cp12* under changing CO_2_ conditions. Growth of Δ*cp12* was not affected at constant light under different inorganic carbon conditions, however, the addition of glucose inhibited growth in darkness as well as under diurnal conditions. The growth defect in the presence of glucose is associated with the inability of Δ*cp12* to utilize external glucose. These phenotypes could be complemented by ectopic expression of the native CP12 protein, however, expression of CP12 variants with missing redox-sensitive cysteine pairs only partly restored the growth with glucose. These experiments indicated that the loss of GapDH-inhibition *via* CP12 is more critical than PRK association. Measurements of the NAD(P)H oxidation revealed an impairment of light intensity-dependent redox state regulation in Δ*cp12.* Collectively, our results indicate that CP12-dependent regulation of the CBC is crucial for metabolic adjustment under conditions leading to redox changes such as diurnal conditions, glucose addition, and different CO_2_ conditions in cyanobacteria.

## Introduction

Cyanobacteria are the only prokaryotes performing oxygenic photosynthesis. This process evolved about 3 billion years ago in ancient cyanobacteria and was later conveyed *via* endosymbiosis into a eukaryotic host cell giving rise to the evolution of all eukaryotic phototrophs, i.e. algae and plants (Hohmann-Marriott and Blankenship, 2011). The light energy captured by photosynthetic complexes is first converted into NADPH and ATP, which are used to energize CO_2_ conversion into organic carbon. All oxygenic phototrophs use the Calvin-Benson cycle (CBC) for CO_2_ fixation. Cyanobacteria evolved an efficient inorganic carbon-concentrating mechanism, which supports CO_2_ fixation *via* ribulose 1,5-bisphosphate carboxylase/oxygenase (RubisCO) under limiting and fluctuating CO_2_ conditions (reviewed in Hagemann et al., 2021), which is active in the light and becomes inactivated in the dark *via* a phytochrome-sensing mechanism (Oren et al., 2021). During the past years, cyanobacteria received much attention as so-called green cell factories, which can also be used to develop sustainable CO_2_-neutral biotechnology to produce energy and feedstock (e.g., Hagemann and Hess, 2018; Appel et al., 2020; Liu et al., 2022).

Photosynthetic light processes and CBC activities need to be coordinated in the diurnal light/dark cycle in all oxygenic phototrophs. This coordination is regulated at different layers and includes mainly redox-signaling to activate or inactivate specific CBC enzymes. It has been shown that CBC regulation involves the action of the small, intrinsically disordered protein CP12 in almost all cyanobacteria, algae and plants (reviewed in López-Calcagno et al., 2014; Gurrieri et al., 2022). Canonical CP12 proteins are characterized by two cysteine (Cys) pairs, one near the N-terminal and another near the C-terminal part, that can form disulfide bonds, which permits CP12 to bind the two CBC enzymes glyceraldehyde 3-phosphate dehydrogenase (GapDH) and phosphoribulokinase (PRK), respectively. Biochemical and structural analyses showed that first two GapDH tetramers are bound and then two PRK dimers are recruited by four oxidized CP12 molecules (Wedel and Soll, 1998; McFarlane et al., 2019; Yu et al., 2020). The complex is formed under oxidizing conditions, particularly during the night in photosynthetic cells, leading to the inhibition of the two enzymes and the CBC in general. Under reducing conditions, i.e. in the light phase, the complex becomes unstable mainly due to the action of thioredoxin(s) or directly through elevated NADPH levels leading to the release of GapDH and PRK and activation of the CBC (**Suppl. Figure S1**). In addition to disulfide bond formation between the Cys pair at the N-terminal part of CP12, the conserved AWD_VEEL motif of CP12 is involved in the inactivation of PRK activity (reviewed in Gurrieri et al., 2022). Recently, it has been reported that CP12 in cyanobacteria, as well as in *Chlamydomonas*, can be phosphorylated under specific conditions (Wang et al., 2014; Spät et al., 2021), however, how far CP12 phosphorylation contributes to or regulates ternary complex formation is not known.

CP12 proteins have been investigated in different cyanobacterial model strains, which showed that many of the basic features of CP12 function are conserved between cyanobacteria and plants (reviewed in López-Calcagno et al., 2014; Gurrieri et al., 2022). It should be noted that cyanobacteria encode two different GapDH proteins. GapDH1 (in *Synechocystis* sp. PCC 6803 encoded by *slr0884*) can only use NAD(H) and is involved in glycolytic carbon catabolism, whereas GapDH2 (in *Synechocystis* sp. PCC 6803 encoded by *sll1342*) can use both, NAD(H) and NADP(H), and is specifically involved in the CBC (Koksharova et al., 1998; own non-published results). Ternary CP12-GapDH2-PRK complex formation under oxidizing conditions has been shown *in vitro* for *Synechocystis* sp. PCC 6803 (Wedel and Soll, 1998) and *Synechococcus elongatus* PCC 7942 (Tamoi et al., 2005) by biochemical measurements such as size-exclusion chromatography (SEC). In the latter strain, the CP12 encoding gene was inactivated and the corresponding mutant showed slower growth under diurnal conditions supporting the *in vivo* role of the CP12 complex formation for coordination of photosynthetic activities in light/dark cycles (Tamoi et al., 2005). Very recently, a *cp12* mutant of *Synechocystis* sp. PCC 6803 which showed severe growth changes in the presence of glucose has been analyzed. Furthermore, distinct redox changes were observed in the absence of CP12, which were used to improve the photosynthetic production of terpenes in this biotechnological chassis (Blanc-Garin et al., 2022). In addition to the canonical CP12 proteins that are conserved in structure and function between cyanobacteria and eukaryotic phototrophs, many cyanobacterial strains harbor genes encoding for CP12-like proteins (Stanley et al., 2012). The CP12-like protein from *Microcystis aeruginosa* PCC 7806 has been analyzed on structural and biochemical levels, which showed that it cannot establish the ternary complex known from canonical CP12 proteins but can inhibit PRK activity in an AMP-dependent manner (Hackenberg et al., 2018). A CP12-like protein from *Synechococcus* sp. PCC 7002 was also not able to complement the phenotypic changes of the *Synechocystis* sp. PCC 6803 *cp12* mutant (Blanc-Garin et al., 2022).

Several hints exist that CP12 also regulates CBC flux and possibly other functions under conditions associated with redox changes (e.g., López-Calcagno et al., 2014). Therefore, we aimed to analyze the impact of CP12 under different growth conditions in *Synechocystis* sp. PCC 6803 (hereafter *Synechocystis*). Using mutants expressing GapDH and PRK tagged with a fluororescent protein allowed us to investigate the dynamics of CP12-dependent CBC regulation for the first time *in vivo*. Fluorescence microscopy verified that GapDH or PRK complexes do not appear in our Δ*cp12* mutant, which also showed distinct metabolic changes such as over-accumulation of ribulose 1,5-bisphosphate (RuBP) and dihydroxyacetone phosphate (DHAP), the products of GapDH and PRK. The mutant had difficulties to grow in the presence of glucose under diurnal and dark conditions, because it showed strongly reduced glucose utilization compared to wild type (WT). These phenotypes could be complemented by ectopic expression of the native CP12 protein, however, expression of CP12 variants with missing redox-sensitive Cys pairs only partly restored the growth with glucose. Furthermore, measurements of the NAD(P)H oxidation kinetic in mutant Δ*cp12* showed an inability to regulate it properly according to the external light intensities compared to WT cells. Collectively, our results indicate that CP12-dependent regulation of the CBC is crucial for metabolic adjustment under conditions leading to redox changes such as diurnal conditions, glucose addition, and different CO_2_ conditions in cyanobacteria.

## Material and methods

### Molecular cloning and mutant generation

For all experiments, the glucose-tolerant wild-type strain of *Synechocystis* sp. PCC 6803 was used (all strains and mutants are listed in **Suppl. Table S1**). The construct for complete deletion of *cp12* in *Synechocystis* was assembled in pJET1.2 (Thermo Fisher Scientific). A fragment consisting of the *cp12* gene with added *Sal*I and *Nde*I restriction sites and the respective adjacent gene flanks was obtained by PCR from genomic *Synechocystis* WT DNA and ligated into the vector. The *cp12* sequence was cut out entirely *via* the added restriction sites and replaced by a kanamycin resistance cassette derived from pUC4K. The complementation constructs were assembled in pVZ322 (Zinchenko et al., 1999). The *cp12* gene variants with adjacent putative promoter sequences were synthesized (BaseGene BV, Leiden, The Netherlands; **Suppl. Figure S2**) and ligated into the vector *via Pst*I and *Xho*I sites. The gentamycin resistance cassette on pVZ322 was cut out *via Sac*I and replaced by a spectinomycin resistance derived from pUC4S. The *cp12* deletion construct was introduced into *Synechocystis* WT cells by direct transformation. The complementation constructs were transferred to Δ*cp12 via* conjugation. All constructs were verified by sequencing. Schematics of plasmid constructs are provided in **Suppl. Figure S3**.

To generate PRK-eYFP and GapDH-eYFP tagged strains, plasmids were synthesized (Genscript, New Jersey, USA) that harbored the following elements: 250 bp upstream of the C-terminal end of the gene of interest without the STOP codon, *Bam*HI and *Eco*RI restriction sites, and 250 bp downstream of the site of insertion in a pUC57-Simple backbone. In a separate plasmid, the tag containing a linker region with a TEV cleavage site and a 6xHis-tag were cloned into the pRSETA-Cr_FDX1-GST-His expression vector (Boehm et al., 2016). The resulting vector was opened with *Nhe*I/*Sma*I and the amplified eYFP was inserted *via* Gibson cloning. Subsequently, the gentamycin antibiotic resistance cassette was amplified and inserted into the *Eco*RV cut vector *via* Gibson cloning. Both plasmids were then cut with *Bam*HI and *Eco*RI resulting in an opened plasmid with the target regions and a cutout fragment containing the tags. These were ligated resulting in the final plasmid transformed into *Synechocystis* to create the eYFP-tagged strains. A list of all primers that were used in this study is provided in **Suppl. Table S2**.

### Growth experiments

Cells of *Synechocystis* were pre-cultivated in shaking 250 ml Erlenmeyer flasks under ambient air conditions at 30°C in buffered BG11 medium (Rippka et al., 1979; TES pH 8.0) with respective antibiotics (mutant Δ*cp12* with 50 µg ml^-1^ kanamycin, complementation strains with 50 µg ml^-1^ kanamycin and 40 µg ml^-1^ spectinomycin, YFP-tagged strains with 5 µg ml^-1^ gentamycin) at a light intensity of 100 µmol photons m^-2^ s^-1^ until an optical density at 750 nm (OD_750_) of 0.8 to 1.0. Cells were then harvested by centrifugation and resuspended in fresh BG11 medium for an acclimation of 24 h in the light. These precultures were then diluted to the desired starting OD_750_, which depended on the growth condition during the experiment. Experiment cultures were grown in 50 ml BG11 in shaking 100 ml Erlenmeyer flasks. For monitoring growth, samples of 1 ml were taken daily from the flasks to measure OD_750_ using the spectrophotometer Genesys 10S UV-Vis (ThermoScientific).

### Metabolite analysis

Cells of *Synechocystis* WT and Δ*cp12* were pre-cultivated for one week in glass tubes bubbling in either low CO_2_ (ambient air with 0.04% CO_2_, LC) or high CO_2_ (5%, HC) conditions in buffered BG11 medium with respective antibiotics at 100 µmol photons m^-2^ s^-1^. Cells were then harvested by centrifugation and resuspended in fresh BG11 medium to OD_750_ of 1.0. After 24 h of acclimation, cultures were adjusted to OD_750_ of 1.0 with fresh BG11. To start the experiment, these cultures were kept growing for 3 h in their respective CO_2_ condition. To shift cultures from HC to LC, cells were harvested by centrifugation and resuspended in equal volume of fresh BG11, then connected to ambient air aeration. For shifts from LC to HC, cultures were directly switched to HC bubbling. Right before, as well as 1 h and 3 h after the respective CO_2_ shifts, four samples of 8 ml were harvested with glass pipettes per culture and quenched directly in 16 ml 70% methanol cooled by dry ice. Quenched cells were pelleted by centrifugation (7 min, 10000 g, 4°C), supernatants were discarded, and cell pellets were quickly frozen in liquid nitrogen and stored at -80°C until extraction. For metabolite extraction, quenched cell pellets were resuspended in -20°C cold 47% methanol/chloroform (5:1, v/v), followed by four cycles of freezing suspensions in liquid nitrogen, 1 h storage in -80°C and thawing on ice. Extracts were lyophilized (Christ Alpha 2-4 lyophilizer, Christ, Germany), resuspended in 250 µl water and filtered (MultiScreen filter plate with Ultracel-10 membrane, Millipore). RuBP and DHAP were quantified by liquid chromatography linked to tandem mass spectrometry (LC-MS/MS) using reverse phase LC (Arrivault et al., 2009).

### Glycogen and glucose quantification

Cellular glycogen was determined according to Gründel et al. (2012) with modifications described by Lucius et al. (2021). During growth experiments, two 5 ml samples of cells were harvested from the flasks and pelleted by centrifugation. The pellet was resuspended in 300 µl 30% KOH (w/v) and then incubated at 95°C for 2 h. After adding 900 µl ice-cold pure ethanol, samples were incubated over night at -20°C. For glycogen precipitation, samples were first centrifuged (10 min, 10000 g, 4°C) and pellets then washed with 1 ml absolute and 1 ml 70% ethanol. The washed pellets were then dried at 50°C. Dry pellets were resuspended in 200 µl sodium acetate buffer (100 mM, pH 4.5) containing 21 U amyloglucosidase and incubated for enzymatic digestion of glycogen at 60°C for 90 min. After centrifugation (10 min, 10000 g, RT), glucose in supernatants was determined using o-toluidine reagent. Glucose contents were calculated using a glucose calibration curve. For quantification of glucose in the medium, two 1 ml samples of culture per time point were harvested during growth experiments. After centrifugation (10 min, 5000 g), the glucose in supernatants was directly determined using o-toluidine reagent.

### Glucose 6-phosphate dehydrogenase activity

Activity of glucose 6-phosphate dehydrogenase (Zwf) was determined in *Synechocystis* crude protein extracts. For light samples, cultures cultivated photoautotrophically in constant light were used, while dark samples were collected after incubation in darkness for three hours. For protein extraction, cells from 15 ml of liquid culture at an OD_750_ of 1 were harvested by centrifugation (10 min, 3000 g, 4°C) and pellets were frozen for 1 h at -20°C. The pellet was then resuspended in 400 µl Tris-HCl buffer (100 mM, pH 7.4). Cells were homogenized by sonication and the lysate was cleared by centrifugation (20 min, 3000 g, 4°C). The protein concentration in the supernatant was determined using Bradford reagent. The Zwf enzyme activity assay was carried out in 96-well plates with a total volume of 200 µl per well. Each enzymatic test was conducted in protein extraction buffer containing 10 µg protein extract and final concentrations of 1 mM NADP^+^ and 5 mM glucose 6-phosphate as substrate at 30°C. To mimic reducing conditions, dithiothreitol (DTT, final concentration 5 mM) was added to selected assays as reducing agent, while others were not treated with DTT. The baseline at 340 nm was measured for 10 min before adding the substrate and monitoring changes in OD_340_ for 30 min. The Zwf activity was calculated from 10 min of linear increase of OD_340_ equivalent to NADPH evolution during the enzymatic reaction.

### Microscopy

To prepare cells for microscopic analyses, they were usually pre-cultivated for up to 5 days in shaking flasks in BG11 medium containing respective antibiotics at 28°C and 100 µmol photons m^-2^ s^-1^ constant illumination. For experimental cultures, cells were transferred to glass tubes and inoculated with fresh BG11 to an OD_750_ of 0.15. Then the strains were cultivated at 28°C and 100 µmol photons m^-2^ s^-1^ constant illumination with ambient air bubbling for three days.

**Figure 1:**
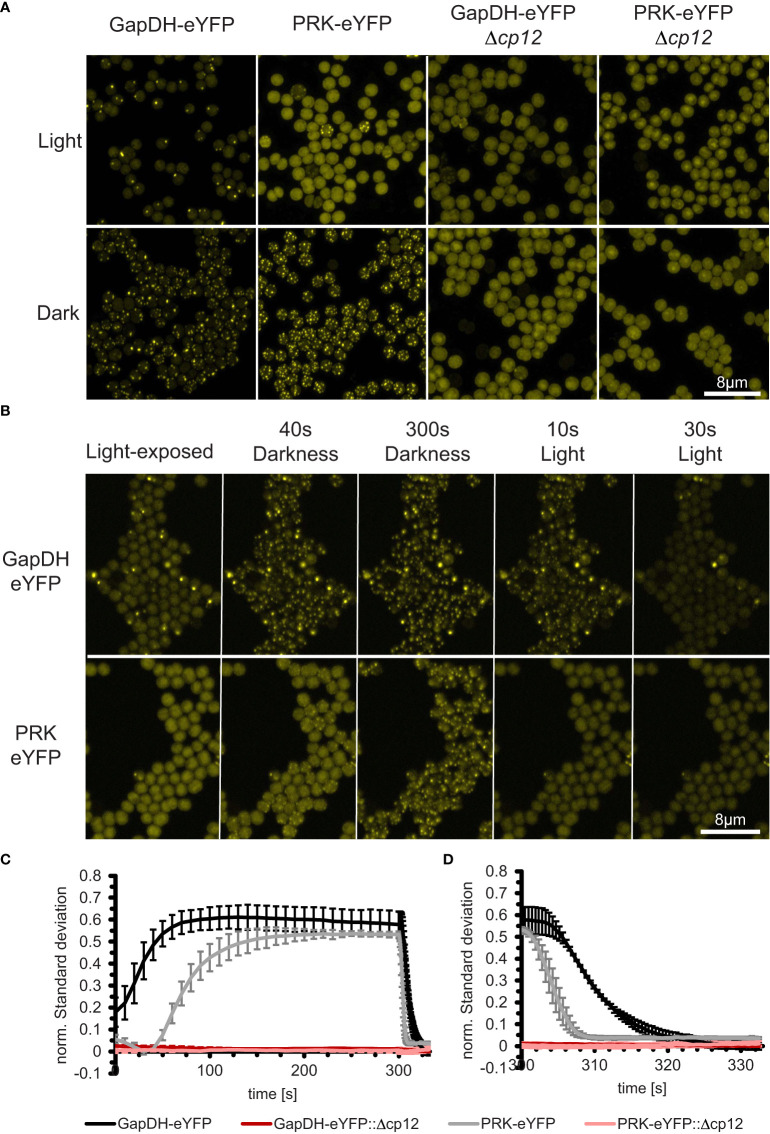
*In vivo* dynamics of CP12-dependent localization changes of the CBB enzymes GapDH2 and PRK in living *Synechocystis* cells during light/dark transition. **(A)** Fluorescence micrographs of *Synechocystis* cells that express GapDH2 or PRK tagged with eYFP in wild type (left two panels) or in Δ*cp12* mutant background (right two panels). All cells were fixed either in light or dark conditions before observation. **(B)** Light-exposed GapDH2-eYFP and PRK-eYFP strains were observed over 300 s after transferring into dark and subsequently 30 s into light conditions, respectively (selected time points are shown). **(C, D)** Kinetics of complex appearance **(C)** or disappearance **(D)** were estimated by plotting the time-dependent change in normalized standard deviations representing the heterogeneity of fluorescence signal distribution inside the cells after transfer from light into darkness **(C)** or back into light **(D)**.

For live cell imaging, 10 µl of cell suspension were dropped on BG11-containing agar plates and incubated at 28°C in darkness for 1 h to let excess liquid evaporate. Next, a BG11 agar cylinder (Ø 1cm) was cut out with a 3D-printed cutter and placed in reverse on a coverslip. This coverslip was placed in reverse on a purpose-built stainless-steel slide (26 mm x 76 mm x 10 mm stainless-steel block with a centered 20 mm hole and a glass slide as cover). The coverslip was fixed with standard petroleum jelly on the stainless-steel slide. The stainless-steel slide was engineered by the research group of Conrad W. Mullineaux (Queen Mary University of London, UK). The slide was modified and manufactured in the in-house University workshop (Christian-Albrechts-University of Kiel, Germany). All experiments were done using an LSM-880 confocal laser scanning microscope (Zeiss, Germany) equipped with a 63x oil immersion objective. Living cells were excited using the 514 nm line of an Argon laser, while fluorescence was detected from 516 – 579 nm for the eYFP channel and 650 – 695 nm for chlorophyll fluorescence (**Figure 1**). Pinhole size was set to 2 airy units (eYFP channel) while laser power, detector gain and scan speed were adjusted to achieve fast scan speeds and decent image quality (equal for all images taken; Metadata available at **Suppl. Data 1**). Fast scan speed and low laser power were essential to limit photoactive illumination. Cells were first illuminated using the bright-field lamp at 15% intensity (~100 µmol photons m^-2^ s^-1^) for 5 min to set them into a photosynthetically-active state. Then, the image sequence was started by switching off the brightfield illumination and one image was taken every 10 s during darkness (illumination time per cell around 50 ms every 10 s). After 300 s, the brightfield illumination was switched on and a new image sequence was started, taking one picture every 0.47 s.

For investigating PRK-eYFP and GapDH2-eYFP distribution at different illumination levels, cells were fixed at precisely defined light intensities. In these experiments, cells were cultivated as described above, harvested after 3 days from glass tubes, washed with fresh BG11, and adjusted to OD_750_ of 1 using BG11. After measuring the NAD(P)H oxidation rate (see 2.8) at one defined light intensity, 25% glutaraldehyde was added to a final concentration of 2%. Illumination conditions were kept constant for 5 min until all cells had been fixed properly. Then, cells were prepared for microscopic analysis as described above. Microscope settings were also similar, but Z-stacks ranging through the single cell layer in 0.38 µm steps were taken (Metadata available at **Suppl. Data 1**).

For High-resolution images of PRK-eYFP and GapDH2-eYFP, cells were fixed either at constant light of 100 µmol photons m^-2^ s^-1^ illumination or in complete darkness. For microscopy the Airy-Scan detector was used (**Figure 1**). To measure the eYFP channel, the 488 nm line of an Argon laser and a double band-path filter 420 - 480 nm and 495 – 550 nm was used, while the chlorophyll fluorescence was detected using a 633 nm laser-diode and a 570 – 620 nm band path + 645 nm long-path filter (Metadata available at **Suppl. Data 1**). Each channel was detected as a Z-stack ranging through the single cell layer in 0.15 µM steps. Finally, Airy-Scan data were processed and transformed into common Z-Stacks using Zen-Software (Zeiss, Germany).

### Image analysis

Live cell images were processed by first concatenating the dark and light stacks in ImageJ (Schindelin et al., 2012) before splitting stacks into stacks of eYFP and chlorophyll fluorescence images. Next, Z-stack max projections were performed on the chlorophyll stacks, creating single-layer pictures. These images were used in an ilastik instance (Berg et al., 2019) trained to distinguish cells from the background creating probability masks used in ImageJ to create regions of interest (ROI) for each cell. These ROIs were used to measure the mean fluorescence intensity and the standard deviation of the eYFP signal in each cell on each image in a stack. The standard deviation was then divided by the mean fluorescence intensity to calculate the normalized standard deviation, representing the heterogeneity of the signal distribution (Mahbub et al., 2020). Pictures of cells fixed at different light intensities were analyzed similarly. The Z-Stacks were converted to a single two-channel image using max Z-projection tool in ImageJ prior to analysis.

### NAD(P)H measurements

*In vivo* NAD(P)H redox dynamics were analyzed using the NAD(P)H module in combination with the Dual-KLAS-NIR system (Walz, Germany). Cultivation of cells was done as described before for microscopic analysis (see 2.6). Cells from 50 ml culture were harvested by centrifugation (4000 g for 3 min), washed twice with BG11-0 (BG11 without nitrogen source), and finally suspended in BG11-0 to an OD_750_ of 1. Cells were kept in 50 ml tubes at 28°C and 100 µmol photons m^-2^ s^-1^ until measured for up to 3 h and then transferred into darkness 30 min before each measurement. 1.25 ml cell suspension were filled into a quartz cuvette placed into the Optical Unit ED-101US/MD connected to the Dual-KLAS-NIR system. Cells were incubated for 3 min during constant stirring at defined light intensities to let the cells acclimate to these conditions before the NAD(P)H oxidation rates were measured four times. One of the two actinic LED arrays was disabled to achieve a lower illumination level. A slow-kinetic trigger run was used containing a 1 s phase with the light intensity investigated, 600 ms high light pulse (3200 µmol photons m^-2^ s^-1^) and 13.4 s darkness. Between each measurement, cells were incubated for 30 s at the investigated light intensity. The last three of the four measured kinetics were averaged, exported and analyzed using R in R-Studio (RStudio Boston, USA). The photo-reducible NAD(P)H level was calculated by subtracting the value at the end of the dark period from the whole measurement and dividing the maximum value between 1 s and 1.6 s by the NAD(P)H level from 0 s to 1 s. The oxidation rates were determined by fitting an exponential function to the measurement from the beginning of the dark period at 1.6 s until the slope was less than 5*10^-8^.

### Protein analysis and Western-blotting

Whole cell extract from *Synechocystis* strains were generated by glass bead breakage as described (Appel et al., 2020). The amount of broken cells was estimated by measuring Chlorophyll a in 2.5 µl of whole cell extract, which was dissolved in methanol to a final volume of 1 ml. Then, the absorption at 666 nm was measured to calculate the chlorophyll a content. Proteins were separated on 10% (w/v) denaturing SDS-Bis/Tris gels using an MES running buffer and Western-blotting were performed as described by Appel et al. (2020). Primary antibodies used in this study were Mouse anti Penta Histidine (Bio-Rad Laboratories, California, USA), Mouse anti Green Fluorescent Protein (Bio-Rad Laboratories, California, USA), and Peroxidase AffiniPure Rabbit Anti-Mouse IgG + IgM (Jackson ImmunoReasearch Europe, Ely, UK).

## Results

### Absence of CP12 prevents *in vivo* complex formation with GapDH and PRK

To analyze the impact of CP12 on carbon metabolism in the model cyanobacterium *Synechocystis*, we generated a completely segregated mutant Δ*cp12.* Deletion of the native *cp12* gene (*ssl3364*) was achieved by replacing the entire coding sequence of CP12 with a kanamycin-resistance cartridge. Genotyping by PCR confirmed complete loss of the gene in Δ*cp12* (**Suppl. Figure S4**).

In the past, the appearance of the ternary CP12-GapDH-PRK complex was usually proven by biochemical measurements such as SEC, native gel electrophoresis, plasmon resonance analysis, or FRET-measurements *in vitro* (e.g., Avilan et al., 1997; Wedel and Soll, 1998; Moparthi et al., 2015). We aimed to obtain the first *in vivo* documentation of complex formation in *Synechocystis* using a fluorescence-tagging approach. To this end, the native GapDH2 (Sll1342) or PRK (Sll1525) encoding genes were replaced by constructs, in which their coding sequences were C-terminally fused with eYFP-encoding sequences and expressed from their native promoters. Genotyping revealed that in the case of PRK, the native gene could be completely replaced by the eYFP-tagged copy, while GapDH2 did not fully segregate (**Suppl. Figure S5**). These eYFP-tagged strains expressed the expected fusion proteins (**Suppl. Figure S6**). When grown under photoautotrophic conditions, they showed only minor growth difference compared to the *Synechocystis* WT, which verifies that the eYFP-tagged enzymes were functional in the CBC (**Suppl. Figure S7**). Fluorescence microscopy showed strong eYFP fluorescence dispersed homogeneously in cells exposed to the light, however, after transfer into darkness within 1-2 min, distinct fluorescing spots appeared in the GapDH2-eYfp-tagged as well as in the PRK-eYFP-tagged strains, which disappeared when the cells were re-exposed to light (**Figures 1A, B**).

Interestingly, these eYFP complexes could not be further observed when the *cp12* gene was mutated in the eYFP-tagged strains (**Figure 1**). Videos showing the kinetics of appearance and disappearance of fluorescent tagged complexes are shown in the supplementary data 2. These results show for the first time the CP12-dependent complex formation with GapDH2 or PRK during light/dark transitions inside living *Synechocystis* cells. Furthermore, the speed of complex formation and disappearance was quantified in the eYFP-tagged strains. The results showed that the binding of GapDH2 occurred faster than the binding of PRK (**Figure 1C**), whereas upon re-illumination, first PRK and then GapDH2 are released from the CP12-dependent complexes (**Figure 1D**). The different kinetics are consistent with the sequential order of binding/releasing the two proteins to oxidized/reduced CP12 found e.g. during crystallography (McFarlane et al., 2019).

### Phenotyping of mutant Δ*cp12* and different complementation strains

The applied mutation strategy permitted the use of the *cp12* flanking regions in attempts for the expression of CP12 variants under control of the native *cp12* promoter. To this end, the native *cp12* gene was reintegrated and the entire construct was cloned into the self-replicating plasmid pVZ322 to achieve the complementation strain Δ*cp12::cp12*-WT. Furthermore, *cp12* gene variants were synthesized, in which the codons for the N-terminal Cys pair (Cys19 and Cys29, docking site for PRK), the C-terminal Cys pair (Cys60 and Cys69, docking site for GapDH2), or all four Cys codons were changed towards serine codons, which gave rise to the complementation strains Δ*cp12::cp12*-ΔCysN, Δ*cp12::cp12*-ΔCysC, and Δ*cp12::cp12*-ΔCysNC, respectively.

**Figure 2:**
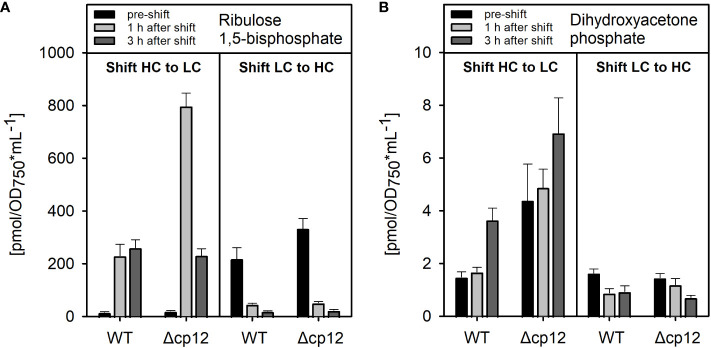
Metabolome analysis of products from the CP12-regulated enzymes PRK and GapDH2. The amounts of dihydroxyacetone phosphate **(A)** and ribulose 1,5-bisphosphate **(B)** were quantified by LC-MS/MS in cells of *Synechocystis* wild type (WT) or mutant Δ*cp12* pre-acclimated to high CO_2_ conditions (5% CO_2_, HC) or ambient air with low CO_2_ (0.04% CO_2_, LC) (black columns – pre-shift). Then, cells were shifted for one (light grey columns) or three hours (dark grey columns) from either HC into LC or LC into HC conditions. Metabolite levels are mean values ± standard deviations from three biological replicates.

First, we compared the growth of the *Synechocystis* WT with mutant Δ*cp12* under different CO_2_ conditions in normal light and observed that both strains grew similarly (**Suppl. Figure S8**). However, metabolome analysis revealed distinct differences in the amounts of ribulose 1,5-bisphosphate (RuBP) and dihydroxyacetone phosphate (DHAP) (**Figure 2**). RuBP is the product of the CP12-regulated PRK reaction, while DHAP is in equilibrium *via* triose-phosphate isomerase with glyceraldehyde 3-phosphate (Gap), the product of CP12-regulated GapDH2 that is chemically unstable and difficult to quantify by LC-MS/MS. RuBP steady state contents were enhanced in mutant Δ*cp12* compared to WT when grown at ambient air (low CO_2_ of 0.04%, LC), while DHAP was higher in mutant cells under high CO_2_ supplementation (5% CO_2_, HC). The differences became more pronounced under transient conditions. Much higher transient accumulations of RuBP and DHAP were observed in mutant Δ*cp12* than in WT 1 h after the shift from HC into LC conditions, while the RuBP contents were almost similar after 3 h HC to LC transfer (**Figure 2**). In contrast to the HC to LC shift, levels of RuBP and DHAP did not differ significantly between the two strains after the transfer of cells from LC into HC conditions. These data indicate that the absence of CP12 had marked influence on *in vivo* PRK and GapDH activities especially under non-steady state HC to LC shift conditions.

**Figure 3:**
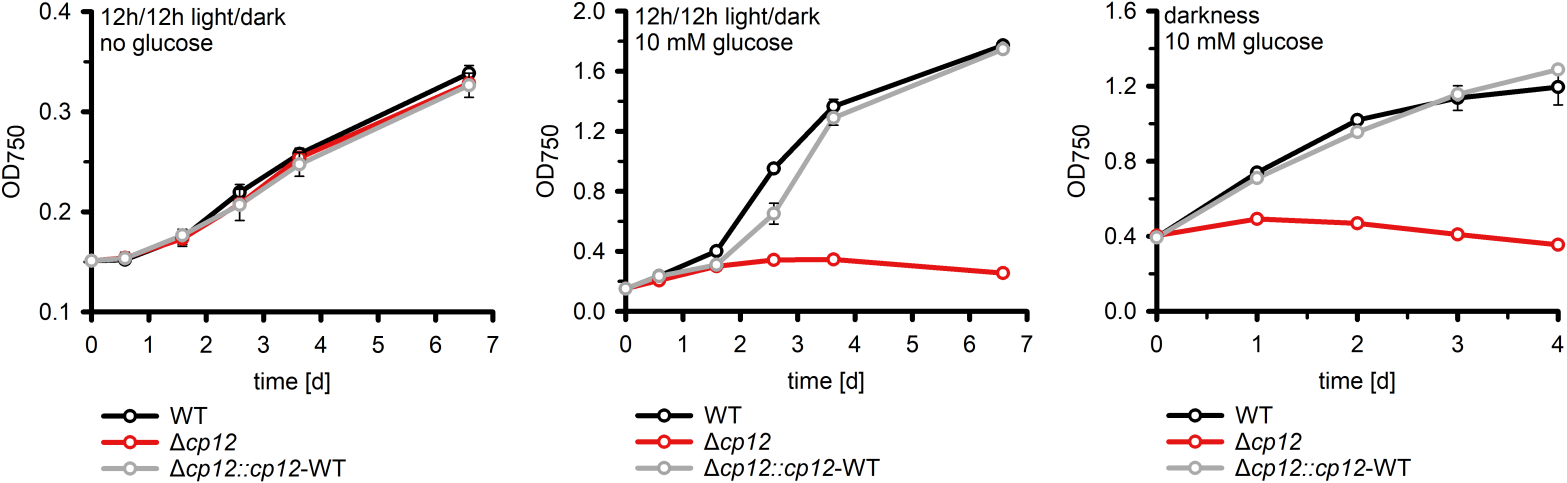
Growth of different strains in the absence and presence of glucose. The increase of optical density at 750 nm (OD_750_) as proxy of biomass was compared in cultures of the *Synechocystis* wild type (WT), the mutant Δ*cp12*, and the complementation strain Δ*cp12::cp12*-WT. Shown are mean values and standard deviations from three independent cultivations.

It has been previously shown that the mutant Δ*cp12* of *Synechococcus elongatus* showed slower growth under diurnal conditions (Tamoi et al., 2005), however, this was not the case with the *Synechocystis* mutant Δ*cp12* (**Figure 3**) as reported recently in an independent study (Blanc-Garin et al., 2022). This different phenotypes could be related to the different culture conditions or to the fact that the CP12 protein in *S. elongatus* is different, i.e. it misses the N-terminal Cys pair. *Synechocystis* is a cyanobacterium that can use external glucose for photomixotrophic growth (Rippka et al., 1979). In the light glucose enters the CBC *via* different glycolytic shunts and thereby enhances the flux through the cycle and CO_2_ fixation (Schulze et al., 2022), which also has marked influence on the cellular redox state (e.g., Wang et al., 2022).

Our mutant Δ*cp12* could grow with glucose supplementation under continuous light (**Suppl. Figure S9**), however, glucose supplementation completely inhibited growth under diurnal conditions and in complete darkness compared to WT (**Figure 3**). The glucose sensitive phenotype of Δ*cp12* was completely reversed to WT-like growth after ectopic expression of native CP12 in the strain Δ*cp12::cp12*-WT (**Figure 3**). Furthermore, *Synechocystis* is capable of so-called photoheterotrophic growth, i.e. the strain can grow on glucose in the light when the photosynthetic electron transport is blocked by the photosystem II-specific inhibitor DCMU (3-(3,4-dichlorophenyl)-1,1-dimethylurea). As found before (Blanc-Garin et al., 2022), the addition of glucose in the light to DCMU-treated cells of mutant Δ*cp12* was toxic, whereas the strain could grow in the absence of DCMU on plates with glucose in continuous light (**Suppl. Figure S9**).

**Figure 4:**
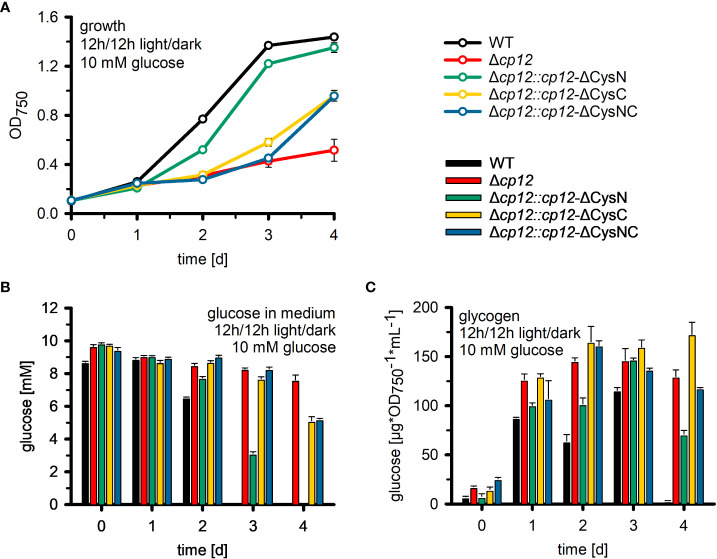
Utilization of glucose in mutant Δcp12 and strains expressing several CP12 variants. **(A)**: The growth as an increase in the optical density at 750 nm (OD_750_) of the *Synechocystis* sp. PCC 6803 wild type (WT) was compared to mutant strain Δ*cp12* with no CP12, and strains expressing CP12 variants with site-specific mutated Cys pairs, i.e. strains Δ*cp12::cp12*-ΔCysN, Δ*cp12::cp12*-ΔCysC, and Δ*cp12::cp12*-ΔCysNC, under diurnal conditions (12 h light/12 h dark) in the presence of 10 mM glucose. Glucose amounts in the medium **(B)** and cellular glycogen amounts **(C)** were quantified daily. Mean values ± standard deviations from three biological replicates are shown.

The CP12 protein is able to interact with two different CBC enzymes. The specific impact of GapDH2 or PRK binding on the observed phenotypic alterations was analyzed in strains expressing CP12 variants either missing the N-terminal Cys pair for PRK association, the C-terminal Cys pair for GapDH2 association, or both. It has been shown that the site-specific mutation of these redox-sensitive Cys residues in CP12 specifically prevents the binding of GapDH2 or PRK (Moparthi et al., 2015). Like the complementation strain with native CP12 (**Figure 3**), the strain expressing the CP12-ΔCysN variant could grow in the presence of glucose in diurnal light/dark cycles almost like WT (**Figure 4A**). In contrast, the strain expressing the CP12-ΔCysC variant initiated slow growth only after the second day, while the lag phase in strain Δ*cp12::cp12*-ΔCysNC was extended to three days (**Figure 4A**). These data indicate that binding and inactivation of GapDH2 activity *via* CP12 has greater impact on glucose sensitivity than the absence of PRK association in strain Δ*cp12::cp12*-ΔCysN.

### Analysis of glucose utilization in mutant Δ*cp12* and different complementation strains

To analyze why the mutant Δ*cp12* could not grow on glucose, the amount of glucose was quantified in the culture medium of different strains. WT cells (**Figure 4B**) and the complementation strain Δ*cp12::cp12*-WT (not shown) consumed all glucose from the medium during the first two days. In contrast, cultures of mutant Δ*cp12* showed only minor glucose consumption. To rule out that a secondary mutation might be responsible for the reduced glucose consumption of mutant Δ*cp12*, the glucose transporter (Sll0771) and glucose-kinase (Sll0593) encoding genes were sequenced revealing that these genes had identical sequences in mutant as in WT (data not shown). Like mutant Δ*cp12*, the strains with mutated C-terminal Cys pair Δ*cp12::cp12*-ΔCysC and Δ*cp12::cp12*-ΔCysNC were strongly affected in glucose consumption. The glucose consumption of strain Δ*cp12::cp12*-ΔCysN was delayed compared to WT but all glucose disappeared after four days, consistent with the ability of this strain to grow in the presence of glucose under diurnal conditions (**Figures 4A, B**).

Glucose addition not only has a marked impact on the redox state of *Synechocystis* cells, the excess organic carbon is also stored as glycogen. Glycogen pools increased in all strains to almost the same extent during the first day after glucose addition (**Figure 4C**). They remained almost stable during the further days in strains Δ*cp12*, Δ*cp12::cp12*-ΔCysC and Δ*cp12::cp12*-ΔCysNC that are not able to grow properly in the presence of glucose in light/dark cycles. In contrast, WT and strain Δ*cp12::cp12*-ΔCysN degraded internal glycogen at the end of the experiment, when the amount of glucose in the medium was consumed or strongly diminished (**Figures 4B, C**). The initial similar ability to accumulate glycogen in all strains indicates that glucose is entering the cells and is channeled into glycogen synthesis independently from CP12, however, the utilization of glucose and later glycogen reserves to promote growth is strongly affected in strains without CP12 or with CP12 variants unable to bind GapDH2.

**Figure 5:**
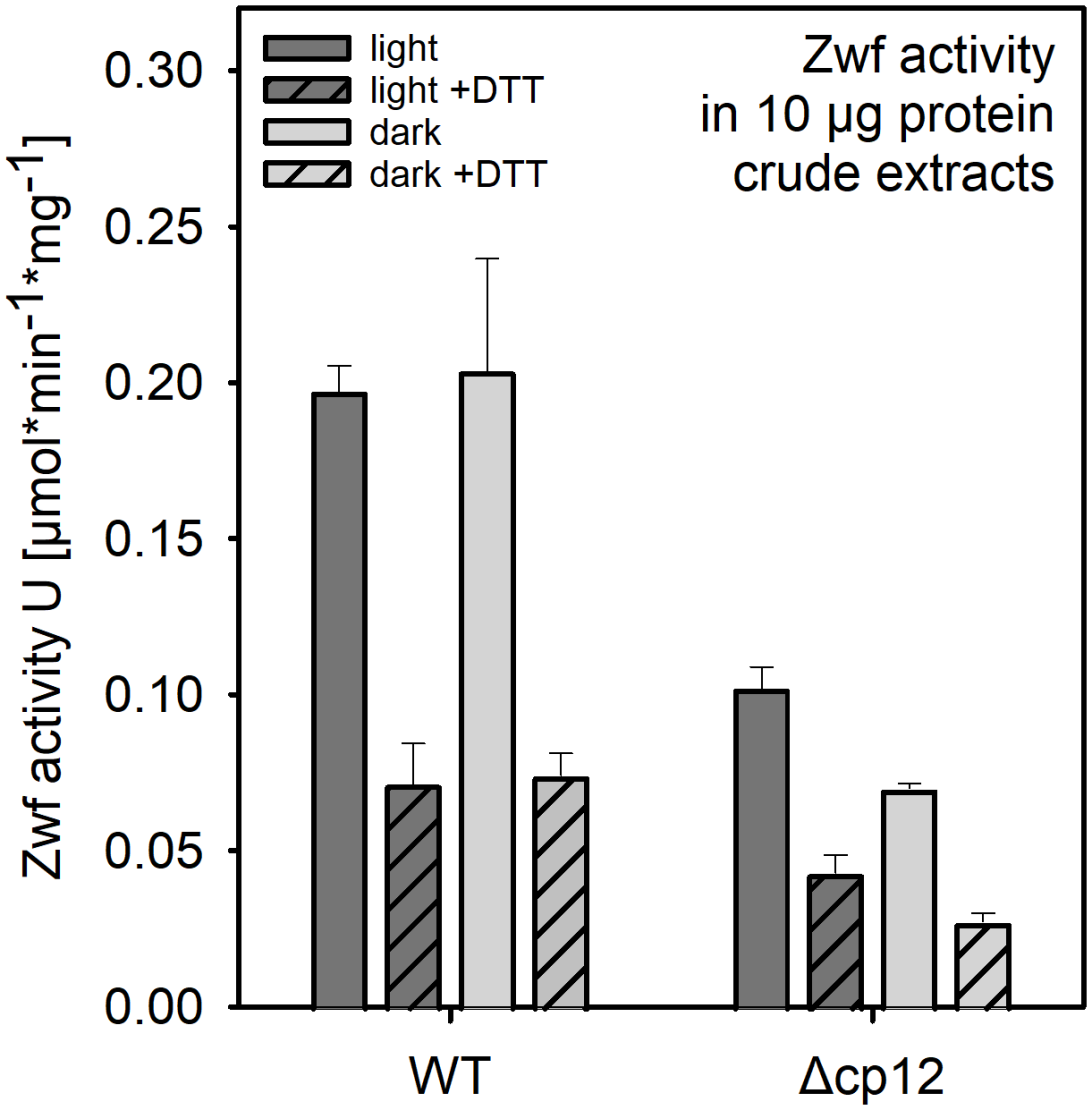
Glucose 6-phosphate dehydrogenase (Zwf) activities in the *Synechocystis* wild type (WT) and mutant Δcp12. Specific enzyme activities were measured in crude protein extracts from cells exposed to light or dark conditions in the presence of saturating glucose 6-phosphate amounts under oxidizing or reducing (+ 5 mM DTT). Data from one representative experiment are shown.

Glucose as well as glycogen catabolism in *Synechocystis* mainly use the oxidative pentose phosphate pathway (OPPP) under dark conditions, which is initiated by the first enzyme glucose 6-phosphate dehydrogenase (Zwf). The activity of Zwf was compared between WT able to use glucose for growth and mutant Δ*cp12* which was not able to do so. Protein extracts were obtained from cells either grown photoautotrophically in the light or from the dark phase. Three-times lower Zwf activities were found in mutant cells in protein extracts obtained from light- or dark-exposed cells (**Figure 5**). It is known that Zwf activity in *Synechocystis* and other cyanobacteria is redox-regulated using the regulator protein OpcA (Sundaram et al., 1998). The redox-regulation of Zwf occurred to almost the same extent in both strains, i.e. addition of DTT decreased the Zwf activity compared to oxidizing conditions to almost 50% (**Figure 5**).

### Analysis of the redox state in mutant Δ*cp12* and different complementation strains

**Figure 6:**
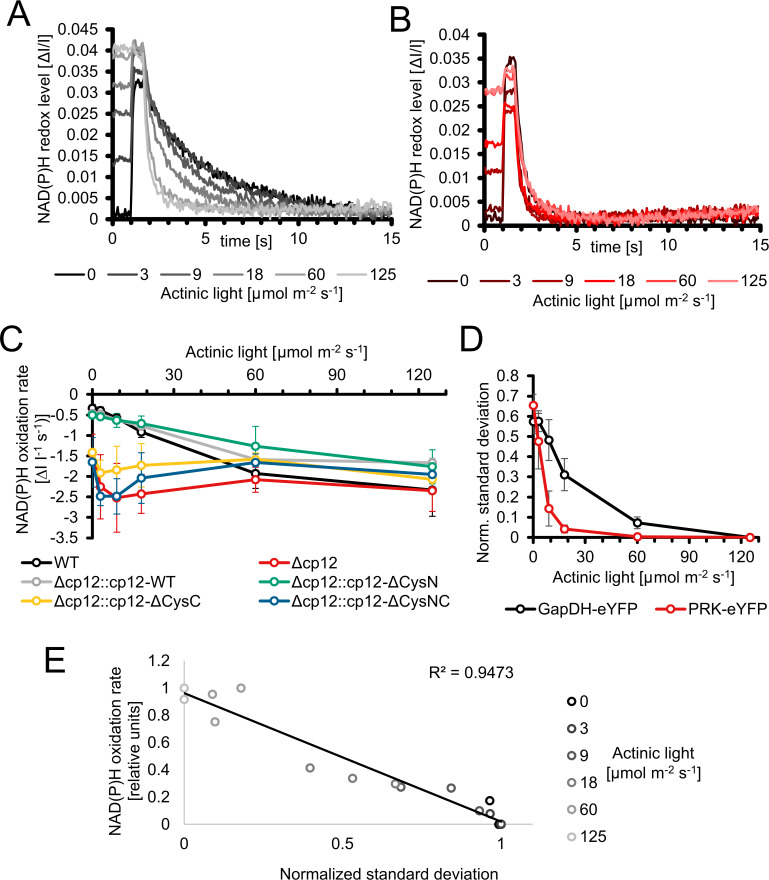
Redox changes in the *Synechocystis* wild type and mutant Δ*cp12* under different light conditions. **(A, B)** NAD(P)H redox changes measured *in vivo via* NAD(P)H fluorescence in wild-type (WT) **(A)** or mutant Δ*cp12* cells **(B)**. The NAD(P)H pool was reduced to its maximum with a strong actinic light pulse (600 ms) and its re-oxidation in darkness was observed. The measured cells were pre-acclimated to the 5 different actinic light intensities or to darkness. **(C)** NAD(P)H oxidation rates of strains expressing different CP12 variants after pre-acclimation to different actinic light intensities. **(D)** Normalized standard deviations representing the heterogeneity of fluorescence signal distribution inside cells. The distribution of PRK-eYFP and GapDH-eYFP is measured under different actinic light conditions. **(E)** Scatterplot of the normalized standard deviation of GapDH-eYFP vs the NAD(P)H oxidation rate under different light intensities.

The action of CP12 on GapDH2 and PRK activities in light/dark cycles is redox-dependent. Moreover, the observed phenotype of mutant Δ*cp12* in the presence of glucose can also result from the inability of proper redox regulation. Redox changes in WT, mutant Δ*cp12*, and in strains expressing different CP12 variants were recorded by measuring NAD(P)H fluorescence changes in cells, which were pre-acclimated to different light intensities. Then, the NAD(P) pool was completely reduced by a saturating light pulse and the re-oxidation of the NAD(P)H was subsequently recorded in darkness. In WT cells, the NAD(P)H pool became re-oxidized to a new steady level within 2-12 s. This re-oxidation process was faster in cells pre-acclimated to stronger light intensities and was slower in cells incubated in darkness or weak light before the saturation pulse (**Figure 6A**). In contrast, the re-oxidation was much faster in cells of mutant Δ*cp12*. Independent from the pre-illumination conditions, it was always completed within 2 s, similar to WT cells exposed to the highest light intensity (**Figure 6B**). This difference indicated that the entire GapDH pool is free in mutant Δ*cp12* and can take active part in the utilization of NAD(P)H, while in WT cells an increasing GapDH pool is bound to CP12 in darkness or under dim light decreasing the re-oxidation time of NAD(P)H. This interpretation is supported by the finding that the re-oxidation kinetics in the mutants with disturbed C-terminal Cys pair for GapDH2 binding, i.e. Δ*cp12::cp12*-ΔCysC and Δ*cp12::cp12*-ΔCysNC showed similarly fast NAD(P)H re-oxidation kinetics as mutant Δ*cp12*. In contrast, the strains expressing native CP12 or the variant with mutated N-terminal Cys pair for PRK binding, i.e. Δ*cp12::cp12*-WT and Δ*cp12::cp12*-ΔCysN are characterized by WT-like NAD(P)H re-oxidation kinetics (**Figure 6C**; **Suppl. Figure S10**). Moreover, the strains expressing eYFP-tagged GapDH2 or PRK in the WT background showed also similar NAD(P)H re-oxidation kinetics as WT (**Suppl. Figure S11**).

The different NAD(P)H re-oxidation kinetics in WT cells after pre-exposure to different light intensities also indicate that part of GapDH2 is already associated with CP12 under low light and less associated under high light, i.e. CP12 is not only mediating CBC activity in light/dark changes but also under different light intensities. This observation initiated attempts to measure the appearance of GapDH2-eYFP or PRK-eYFP complexes in cells exposed to different actinic light intensities under the fluorescence microscope. These measurements show an exponential increase of PRK-eYFP and particularly of GapDH2-eYFP complex fluorescence with decreasing light intensities towards the maximum in complete darkness (**Figure 6D**). Plotting the oxidation rate of the NAD(P)H pool with the normalized standard deviations representing the heterogeneity of the fluorescence signal of GapDH2-eYFP resulted in a linear relation (**Figure 6E**).

## Discussion

It is generally accepted that CP12 can bind GapDH and PRK under oxidative conditions, which occurs especially in darkened cells of oxygenic phototrophs to inactivate the CBC activity when light processes are not further working (reviewed in López-Calcagno et al., 2014; Gurrieri et al., 2022). However, there is an increasing body of evidence that CP12-mediated regulation may extend over the light/dark regulation of CBC activity. To analyze the role of CP12 regulation in the model cyanobacterium *Synechocystis*, we first generated eYFP-tagged strains in *Synechocystis* to monitor for the first time CP12-GapDH2-PRK complex formation *in vivo*. Expressing eYFP-tagged PRK or GapDH2 variants in *Synechocystis* clearly showed the appearance of CP12-dependent complexes in darkness and their disappearance in the light. In the dark-shifted cells highly fluorescent spots appeared, which were not further visible in the mutant Δ*cp12*. A clear correlation between spot appearance and the oxidation rate of the NAD(P)H pool was observed (see **Figure 6**), which indicated that their appearance is redox-dependent and resulted in inactivation of at least the GapDH2. The appearance of a distinct number of obvious large complexes was not expected and could indicate that CBC enzymes might be localized at specific parts of the cyanobacterial cell. One possible location would be near the carboxysome, which harbors all active RubisCO in the cyanobacterium. Usually one to five carboxysomes are visible in electron micrographs of *Synechocystis* (e.g. Hackenberg et al., 2012), which is similar to the number of CP12-GapDH2-PRK fluorescent spots, hence, these complexes might be associated to carboxysome(s) (see **Figure 1**). Instead of eYFP-tagging PRK or GapDH2, truncated versions of the CP12 protein were fused N-terminally with CFP and C-terminally with YFP to monitor thioredoxin-mediated redox-changes in *Anabaena* sp. PCC 7120 or in chloroplasts of Arabidopsis *in vivo via* FRET (Sugiura et al., 2019). These redox-sensing devices showed clear fluorescence increases of YFP under oxidizing conditions, i.e. when the internal disulfide bond was established after transfer into darkness, or decreased YFP fluorescence under reducing conditions, i.e. when the disulfide bond was released *via* reduced thioredoxin(s) after illumination. As found in our tagged strains (see **Figures 1C, D**), the reduction of CP12 *via* thioredoxins in the light occurred faster than its oxidation after transfer into darkness (Sugiura et al., 2019).

Our fluorescent-tagged strains further allowed us to follow the different kinetics of GapDH2 and PRK binding to and release from CP12 in dark/light cycles. As expected from the structural model (McFarlane et al., 2019), GapDH-eYFP complexes appeared faster and disappeared more slowly than the PRK-eYFP complexes. Hence, the fluorescent-tagged *Synechocystis* strains also verified the predicted sequential order of CP12-complex formation *in vivo*. Moreover, the rate of complex formation was followed in darkened cells after their pre-incubation under different light intensities. The different kinetics clearly showed that *in vivo* at lower light, when the photosynthetic activity and hence the CBC flux is diminished, already a substantial part of GapDH2 is bound to CP12, whereas under high light conditions inducing high photosynthetic activities much less GapDH2 is CP12-associated (see **Figure 6**). These results provide clear evidence that CP12 is not only a device to switch on and off CBC activities in diurnal light/dark cycles, but it also fine-tunes CBC activities and light processes during the day under different light and accompanied redox conditions in the *Synechocystis* cell. Differences in the available light, especially high light incubations, have been shown also to induce intracellular fluctuations in the available CO_2_, because many genes encoding proteins for acclimation to low CO_2_ conditions were clearly induced in the transcriptome of *Synechocystis* exposed to high light conditions (Hihara et al., 2001). The observed changes in the amounts of RuBP and DHAP in Δ*cp12* cells under non-steady state conditions in cells switched from HC into LC conditions (see **Figure 2**) provide another indication that the enzymes GapDH2 and PRK are more active in the absence of CP12 compared to WT cells even under continiuous light conditions.

The most severe phenotype in the Δ*cp12* cells was observed when the cells can consume added glucose in darkness as has been recently also reported by Blanc-Garin et al. (2022). These results indicate that in addition to CBC, the redox-dependent CP12-mediated regulation has a broader impact on carbohydrate metabolism than just regulating the CBC. Our results indicate that glucose uptake and glycogen metabolism is not primarily affected, but the glucose catabolism *via* the OPPP is strongly diminished. This hypothesis is supported by the reduced activity of Zwf in cells of Δ*cp12* (see **Figure 5**). The glucose-sensitive phenotype could be explained by the absence of proper GapDH2 and PRK regulation, initiation of futile cycles, and/or more enzymes of primary C-metabolism being regulated by CP12. The latter assumption is supported by the reported association of aldolase, which also plays an important role in sugar metabolism in oxygenic phototrophs, to the CP12-GapDH-PRK complex in *Chlamydomonas* (Erales et al., 2008). However, the main function of CP12-mediated inactivation of GapDH2 and PRK is usually related to the avoidance of futile cycles between CBC and OPPP. The non-oxidative portion of the OPP overlaps to a large extent with the regenerative part of the CBC by the used enzymes but are involved in sugar catabolism and anabolism, respectively (reviewed in López-Calcagno et al., 2014; Gurrieri et al., 2022). In this regard, the proper regulation of GapDH2 activity seems to be more important than PRK regulation, because expressing CP12 variants with mutated C-terminal Cys pairs necessary to bind GapDH2 show much fewer capabilities to complement the glucose-sensitive phenotype of Δcp12 (see **Figure 4**) or the observed redox imbalance (see **Figure 6C**). GapDH2 can use NADPH and is mainly involved in the conversion of glycerate 1,3-bisphosphate into Gap, while the GapDH1 that is supposed to be mostly active under dark conditions in WT cells (Koksharova et al., 1998) catalyzes the reverse reaction in WT cells. Hence, the dual activities of the two GapDH isoenzymes during the night could prevent proper glycolytic breakdown of glucose under dark conditions in Δ*cp12.* Moreover, the utilization of NADPH by GapDH2 can likely also result in redox imbalance, because in WT cells NADPH is mostly produced in the dark *via* the OPPP, which is necessary to fuel important other enzymatic reactions of primary metabolism. Hence, redox imbalances especially in the presence of glucose could be another reason for the inability of Δ*cp12* to grow on glucose. However, in contrast to the independent study by Blanc-Garin et al. (2022) we could not detect glucose sensitivity at constant light. This deviation could be related to the different culture conditions in the two studies. In our experiments we grew cells at higher light intensities than in the experiments reported by Blanc-Garin et al. (2022). Our data in **Figure 6** clearly indicate that at low light conditions part of CP12 is already associated with GapDH2, while this interaction is not detectable at higher light intensities.

Of course, mutation of CP12 could also impact the amount of its binding partners GapDH2 and PRK. Decreased PRK expression has indeed been reported in *cp12* mutants of Arabidopsis, whereas the amount of GapDH and many other CBC enzymes was not changed (López-Calcagno et al., 2017). However, the GapDH2 and PRK activities remained unchanged in another Δ*cp12* mutant of *Synechocystis* (Blanc-Garin et al., 2022). As shown in the previous study and by us, expressing CP12 variants with N-terminal changed Cys pair involved in PRK-binding permits complementation of glucose utilization (see **Figure 4**) and results in WT-like redox changes (see **Figure 6C**). It has been shown that in addition to redox-dependent regulation of PRK *via* the CP12-GapDH complex, PRK activity in cyanobacteria and plants is directly redox-regulated by two internal Cys pairs, which can undergo redox-dependent disulfide bond formation thereby modulating PRK activity. Fukui et al. (2022) recently analyzed the contribution of this redox regulation and showed that especially the C-terminally located Cys pair in PRK is subject to thioredoxin-mediated redox regulation. Oxidation of this Cys pair contributes to decreased PRK activity in the cyanobacterium *Anabaena* sp. PCC 7120, whereas its oxidation supports the binding of PRK into the CP12-GapDH complex in both, the cyanobacterium as well as Arabidopsis (Fukui et al., 2022). Hence, proper PRK regulation is achieved on more layers in cyanobacteria than just CP12, which seems to be the major regulatory measure to regulate GapDH2 and the photosynthetic CBC activity.

Collectively, our results indicate that CP12-dependent regulation of the entire carbon metabolism including the CBC and OPPP is crucial for metabolic adjustment under conditions leading to redox changes. In addition to diurnal conditions, we present evidence that redox changes due to glucose addition, different CO_2_ or light conditions also depend on CP12-mediated metabolic fine-tuning in cyanobacteria. In this regard, the proper regulation of GapDH2 seems to be more important than PRK regulation, because the GapDH isoenzymes catalyze opposite reactions in carbon anabolism and catabolism and also use large amounts of NADPH or NADH, thereby significantly contributing to redox changes under the different growth conditions.

## Data availability statement

The original contributions presented in the study are included in the article/supplementary material. Further inquiries can be directed to the corresponding author.

## Author contributions

MH and KG conceived and planned the experiments. SL constructed mutant strains, performed physiological and biochemical characterization. SH generated fluorescence tagged strains. MT performed fluorescence microscopy and redox measurements. CM contributed to the establishment of fluorescence microscopy and its evaluation. SA processed and analyzed the LC-MS/MS data. MH wrote the manuscript with contributions of all authors. All authors approved the submitted version.

## Funding

MH and KG acknowledge the funding from the German Research Foundation in the frame of the research consortium SCyCode (DFG, HA 2002/23-1, GU 1522/5-1, FOR2816) and from the Universities of Rostock and Kassel. SA was supported by The Max Planck Society.

## Acknowledgments

The technical assistance of Klaudia Michl at the University of Rostock is acknowledged. We thank the Central Microscopy facility at the Department of Biology at the University of Kiel for assistance to establish microscopic analyses of eYFP-tagged strains.

## Conflict of interest

The authors declare that the research was conducted in the absence of any commercial or financial relationships that could be construed as a potential conflict of interest.

## Publisher’s note

All claims expressed in this article are solely those of the authors and do not necessarily represent those of their affiliated organizations, or those of the publisher, the editors and the reviewers. Any product that may be evaluated in this article, or claim that may be made by its manufacturer, is not guaranteed or endorsed by the publisher.
