# Supplementary material for: CP12 fine-tunes the Calvin-Benson cycle and carbohydrate metabolism in cyanobacteria
Source: Front Plant Sci. 2022 Oct 11;13:1028794. doi: 10.3389/fpls.2022.1028794 (PMC9623430; doi:10.3389/fpls.2022.1028794)
Supplement: Supplementary file 1 [file DataSheet_1.pdf]

Supplementary Data of the Frontiers in Plant Science manuscript

CP12 fine-tunes the Calvin-Benson cycle and carbohydrate metabolism in cyanobacteria

Running title: Role of CP12 in *Synechocystis*

Stefan Lucius[1], Marius Theune[2,3], Stéphanie Arrivault[4], Sarah Hildebrandt[3], Conrad W. Mullineaux[5], Kirstin Gutekunst[2,3], Martin Hagemann[1]*

1 - University Rostock, Department Plant Physiology, Albert-Einstein-Str. 3, 18059 Rostock, Germany

2 – University Kassel, Molecular Plant Physiology, Bioenergetics in Photoautotrophs, Heinrich-Plett-Str. 40, 34132 Kassel, Germany

3 - University Kiel, Botanical Institute, Am Botanischen Garten 5, 24118 Kiel, Germany

4 - Max Planck Institute of Molecular Plant Physiology, Am Mühlenberg 1, 14476 Potsdam-Golm, Germany

5 – School of Biological and Behavioural Sciences, Queen Mary University of London, London, United Kingdom

***Corresponding author:** Martin Hagemann, Institut für Biowissenschaften, Abteilung Pflanzenphysiologie, Universität Rostock, Albert-Einstein-Str. 3, Rostock D-18059, Germany; Tel: +49(0)3814986110; Fax: +49(0)3814986112; Email: martin.hagemann@uni-rostock.de

Table S1: List of strains investigated in the present study
Table S2: List of oligonucleotides used in the present study

Figure S1: Schematic view on CP12-mediated redox regulation of GapDH and PRK
Figure S2: Insert sequences for Δ*cp12* complementation constructs
Figure S3: Schematics of plasmids for creation of Δ*cp12* and complementation strains
Figure S4: Genotyping by PCR of Δ*cp12* and complementation strains
Figure S5: Genotyping by PCR of eYFP-tagged strains
Figure S6: Western blot analysis of eYFP tagged stains
Figure S7: Growth of eYFP-tagged strains
Figure S8: Growth of wild type and mutant Δ*cp12* under permissive conditions
Figure S9: Photoheterotrophic growth in the presence or absence of DCMU on plates
Figure S10: Ratio of photo-reducible NAD(P)H under different actinic light intensities
Figure S11: NAD(P)H oxidation kinetics in GapDH-eYFP and PRK-eYFP cells adapted to different light intensities

Supplementary Data 1: Metadata to obtain microscopic images

Supplementary Data 2: Video of PRK-eYFP-His and GapDH2-eYFP-His strains under light-dark and dark-light transition

Supplementary Table S1. List of strains investigated in the present study.

Strain name	Description
Synechocystis WT	*Synechocystis* sp. PCC 6803, glucose-tolerant wild type (WT)
Δ*cp12*	WT with deleted native *cp12* gene *ssl3364,* replaced by Kan^{R} gene *aphII*
Δ*cp12::cp12*-WT	Δ*cp12* with pVZ322 harboring native *cp12* gene and Spec^{R} gene *aadA*
Δ*cp12::cp12*-ΔCysN	Δ*cp12* with pVZ322 harboring *cp12* gene with replaced cysteines C19S-C29S and Spec^{R} gene *aadA*
Δ*cp12::cp12*-ΔCysC	Δ*cp12* with pVZ322 harboring *cp12* gene with replaced cysteines C60S-C69S and Spec^{R} gene *aadA*
Δ*cp12::cp12*-ΔCysNC	Δ*cp12* with pVZ322 harboring *cp12* gene with replaced cysteines C19S-C29S-C60S-C69S and Spec^{R} gene *aadA*
GapDH-eYFP	WT with c-terminal eYFP-tagged GapDH2 (*sll1342*) and Gent^{R} gene
PRK-eYFP	WT with c-terminal eYFP-tagged PRK (*sll1525*) and Gent^{R} gene
GapDH-eYFP::Δ*cp12*	GapDH-eYFP with deleted native *cp12* gene *ssl3364* replaced by Kan^{R} gene *aphII*
PRK-eYFP::Δ*cp12*	PRK-eYFP with deleted native *cp12* gene *ssl3364* replaced by Kan^{R} gene *aphII*

Supplementary Table S2. List of oligonucleotides used in the present study.

Primer name	Sequence 5'-3'	Application
cp12-fw	ATGAGCAATATTCAAGAAAAAAATCGAAC	genotying Δ*cp12* strains
cp12-rv	CTAGTCGTCGTAAATGCGGCAC	genotying Δ*cp12* strains
cp12-flank-fw	GTACAAGGCCGCAATGGTGAG	genotying of Δ*cp12,* GapDH2-eYFP:: Δ*cp12* and PRK-eYFP:: Δ*cp12* and creation of Δ*cp12* strains
cp12-flank-rv	TAGGCGAATTAAGCCAACGTCTGTC	genotying of Δ*cp12,* GapDH2-eYFP:: Δ*cp12* and PRK-eYFP:: Δ*cp12* and creation of Δ*cp12* strains
cp12-fw-SalI	GTCGACATGAGCAATATTCAAGAAAAAAATCGAAC	generation of Δ*cp12* strains
cp12-rv-NdeI	CATATGCTAGTCGTCGTAAATGCGGCAC	generation of Δ*cp12* strains
cp12-Fus-P2	TATTGCTCATGTCGACAAAGTTTAACCGCCTACGATTG	generation of Δ*cp12* strains
cp12-Fus-P3	CGACGACTAGCATATGGGGCTGATGGGGCAAACCC	generation of Δ*cp12* strains
YFP-for	AGGGCGGCGCTAGCATGGTGAGCAAGGGCGAGGAGCTGTT	generation of pRSETA-Cr_eYFP-His
YFP-rev	GTGATGGTGATGGTGATGCCCGGGCTTGTACAGCTCGTCCA	generation of pRSETA-Cr_eYFP-His
YFP-His-GENT-Fw	CACCATCACCATCACTAAGATGTCGACGGATGAAGGCACGAA	generation of pRSETA-Cr_eYFP-His_Gent
GENT-Rev	CAAGCTTCGAATTCCTAGATGTCGACCGAATTGTTAGGTGG	generation of pRSETA-Cr_eYFP-His_Gent
GapDH2-fw	TTAAAGGAGTGTTGGAATACACCGATTTGG	Genotyping of GapDH2-eYFP strain
GapDH2-rv	ATGGGGAAAGTTTCGCCGGG	Genotyping of GapDH2-eYFP strain
PRK-fw	TAGAGGAAATGGTTTATGTGGAAAACCACC	Genotyping of PRK-eYFP strain
PRK-rev	GGCATCATTATCCTCCCCAGAAATAAGC	Genotyping of PRK-eYFP strain

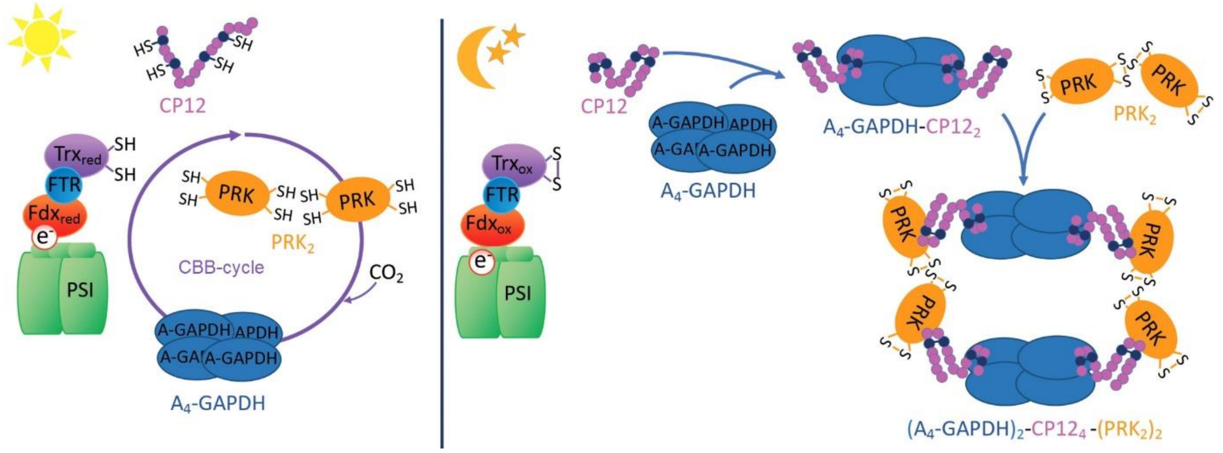

Figure S1: Schematic view on CP12-mediated redox regulation of GapDH and PRK. Under reducing conditions, i.e. in the light, the CP12 is disordered due to the reduction of all four cysteine residues (depicted as dark dots) via thioredoxin (Trx). The two enzymes glyceraldehyde 3-phosphate dehydrogenase (GAPDH) and phosphoribulokinase (PRK) are active and participate in the CO_2-fixing Calvin-Benson-Bassham cycle (CBB cycle). Under oxidizing conditions, i.e. in the darkness, the CP12 is structured due to the disulfide bond formations at the N- or C-terminal cysteine residues, which then permitted to bind GAPDH and PRK thereby inactivation the CBB cycle.

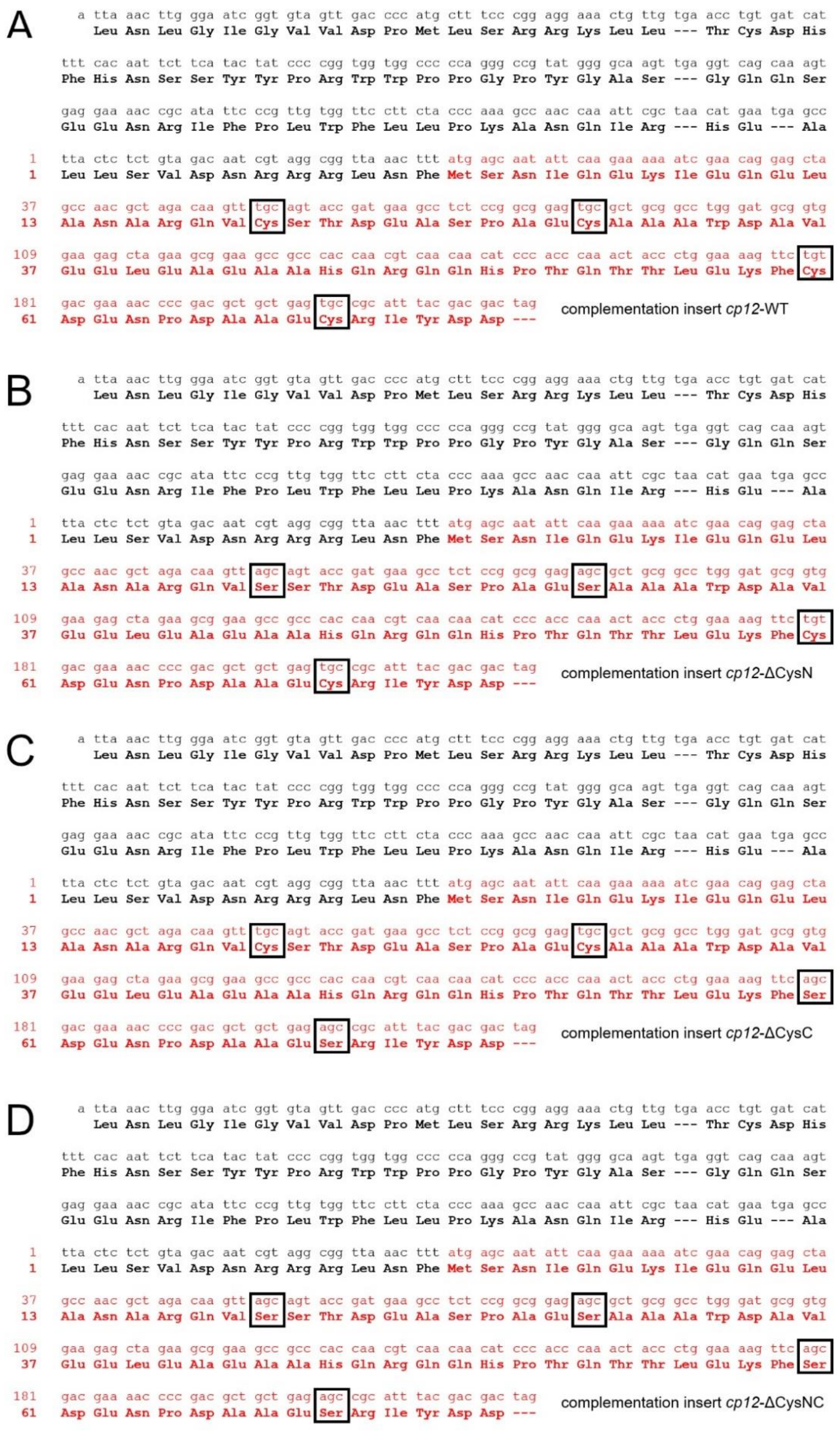

Supplementary Figure S2: Insert sequences for Δ*cp12* complementation constructs. Variants of *cp12* gene sequence have been synthesized and cloned into vector pVZ322. Black boxes indicate locations of cysteines or their replacement for serines, respectively. Black letters mark putative native promoter sequence of *cp12*. Red letters mark *cp12* gene sequence. **A** - native *cp12*. **B** - N-terminal cysteine pair replaced by serines. **C** - C-terminal cysteine pair replaced by serines. **D** - both cysteine pairs replaced by serines.

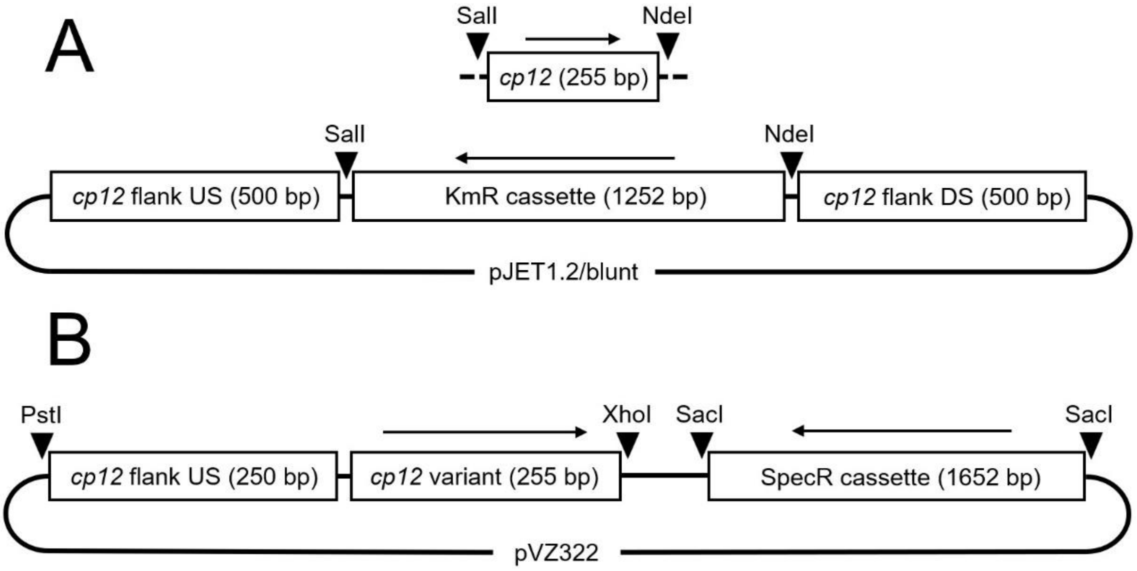

Supplementary Figure S3: Schematic drawings of plasmids for the generation of Δ*cp12* and complementation strains. A: *cp12* deletion mutant construct. **B**: example for generation of *cp12* complementation strains.

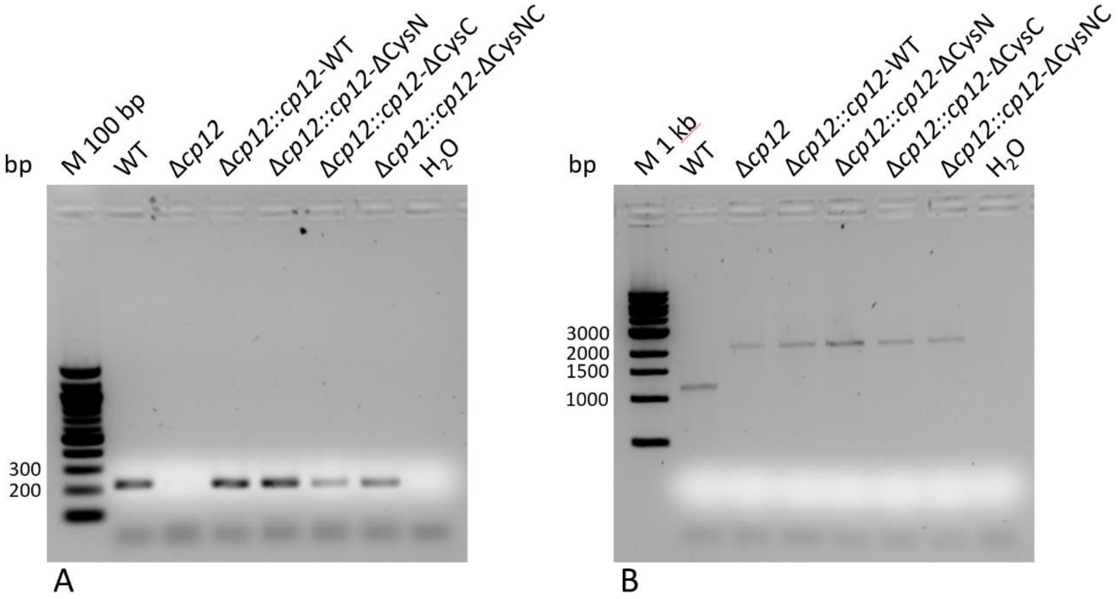

Supplementary Figure S4: Genotyping by PCR of Δ*cp12* and complementation strains. A - amplification with *cp12* gene specific primers cp12-fw/cp12-rv. **B** - amplification with *cp12* gene flanking primers cp12-flank-fw/cp12-flank-rv that bind 500 bp upstream or downstream of *cp12*. DNA from the different strains used for PCR is given for each lane. PCR results confirm complete segregation of all mutant strains.

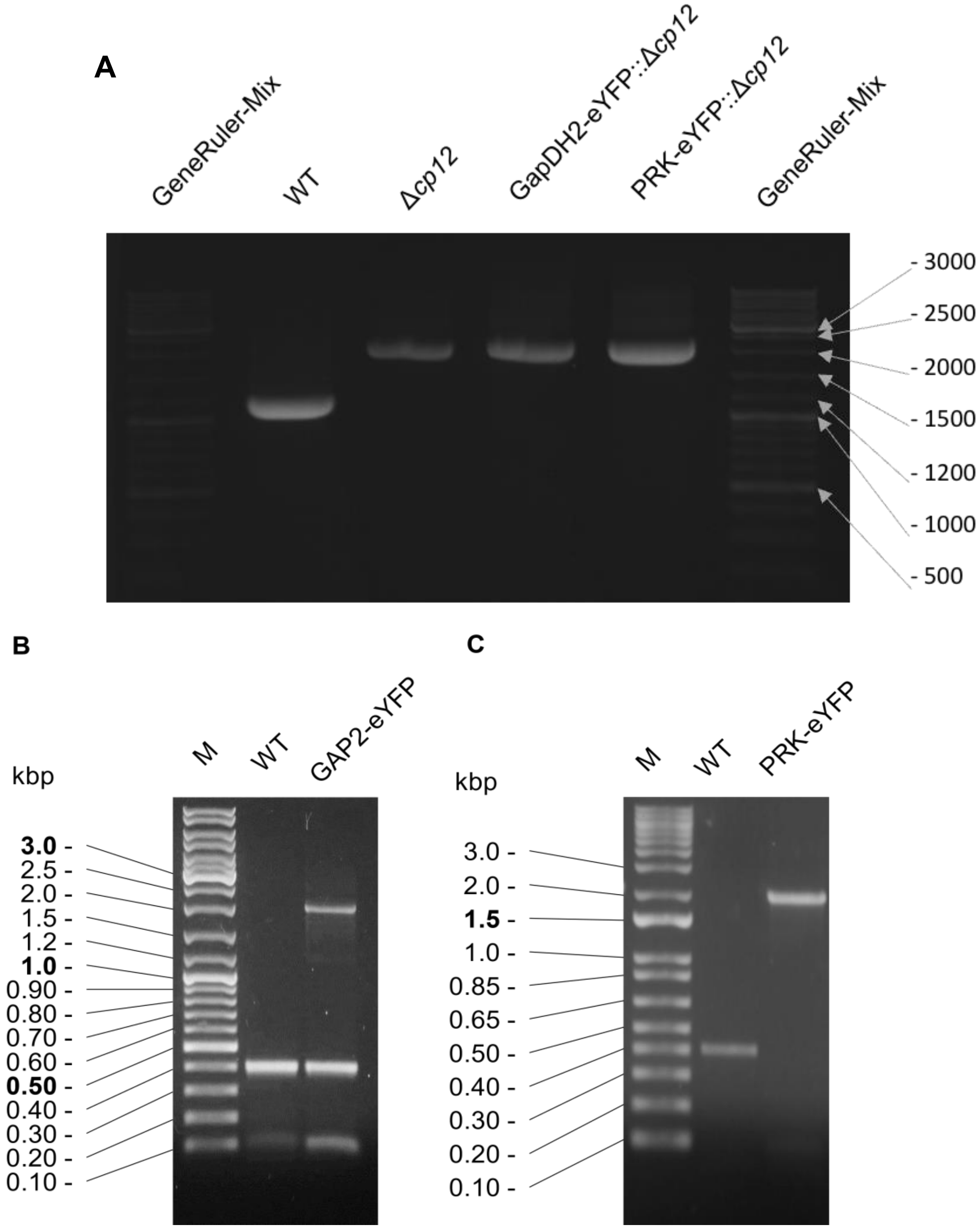

Supplementary Figure S5: Genotyping by PCR of eYFP-tagged strains.
A - Amplification of the *cp12* gene using primers cp12-flank-fw/cp12-flank-rv that bind 500 bp upstream or downstream of *cp12*. **B**- Amplification of the *gapDH2* gene using primers GapDH2-fw/GapDH2-rv that bind 250 bp upstream or downstream of the *gapDH2 gene*. **C**- Amplification of the *prk* gene using primers PRK-fw/PRK-rv that bind 250 bp upstream or downstream of the *prk gene*. The template-DNAs were isolated from different strains as specified above each lane. PCR results confirm complete segregation of all mutant strains except GapDH2-eYFP.

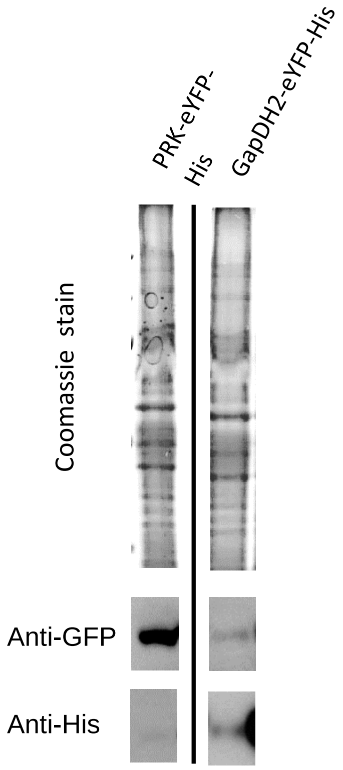

Supplementary Figure S6: Verification of the protein expression of the GapDH2-eYFP-His and PRK-eYFP-His by Western-blotting.

Cells were cultivated under photoautotrophic conditions in BG11. Whole-cell extracts were used to verify that the attached eYFP-His of the respective tagged proteins are expressed and that the fluorophores are bound to the protein of interest. The same amount of chlorophyll (0.15 µg) was loaded to each lane. Anti-GFP and anti_His antibodies showed a signal at the expected sizes: PRK-eYFP-His ~53 kDa; GapDH2-eYFP-His ~68kDa**.**

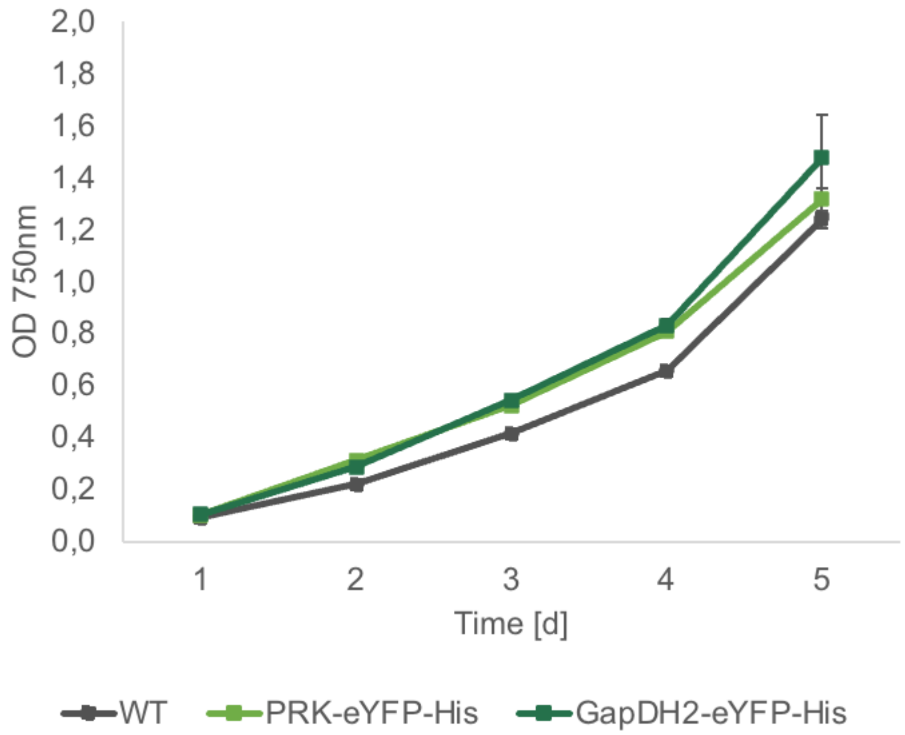

Supplementary Figure S7: Growth of eYFP-tagged strains under standard conditions. Growth of eYFP tagged strains was monitored under photoautotrophic conditions with bubbling by ambient air.

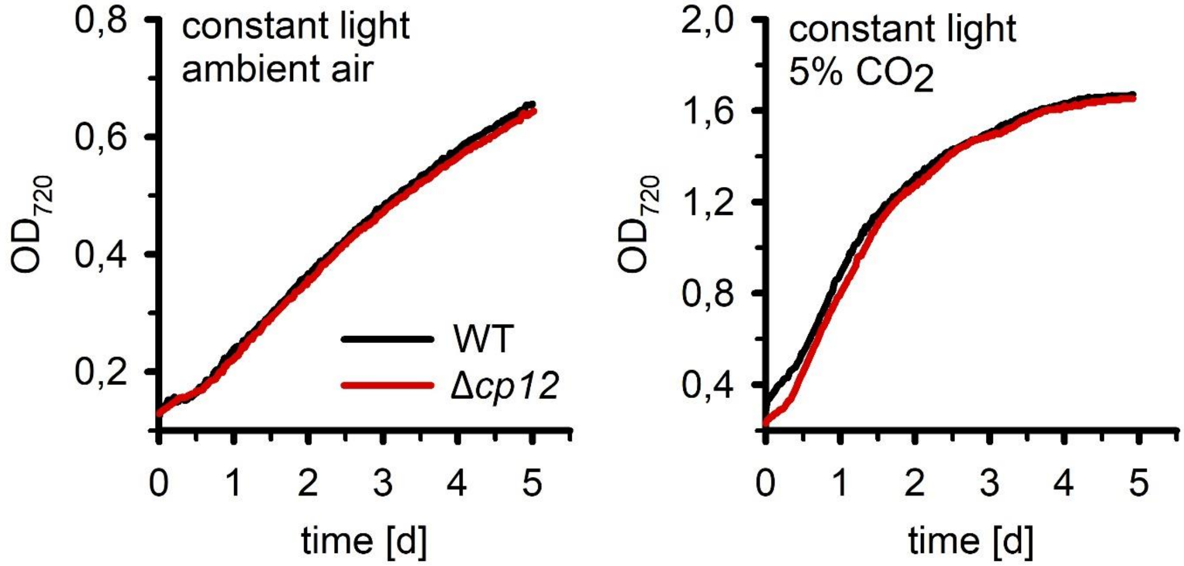

Supplementary Figure S8: Growth of wild type and mutant Δ*cp12* under permissive conditions. Cultures were grown at ambient air (0.04% CO_2, LC) and high CO_2 conditions (5% CO_2, HC). The increase of OD_{720} is displayed compared as proxy of biomass.

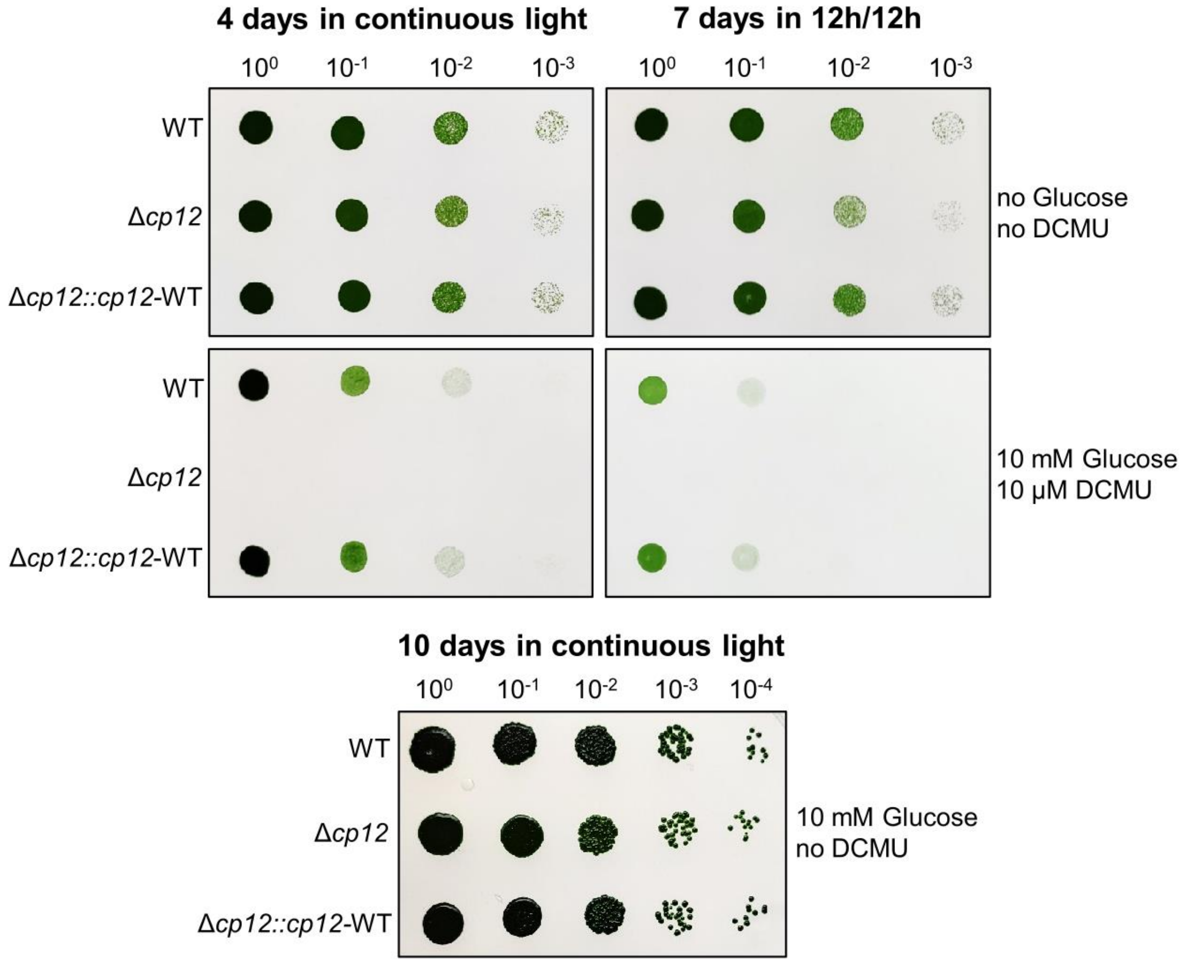

Supplementary Figure S9: Photoheterotrophic growth in the presence or absence of DCMU on plates. Liquid cultures were grown in shaking flasks under photoautotrophic conditions for three days. Samples of 1 ml culture were taken and their OD_{750} was adjusted to 0.7 (dilution 10^0) with fresh BG-11 for the drop dilution assays. A dilution series up to 10^{-4} was created for each strain and drops of 2 µl culture were pipetted in a grid on 1.5% bacto agar BG-11 plates containing combinations of 10 mM glucose and 10 µM DCMU or none of these additions. The plates were then incubated in either continuous light or diurnal conditions for the indicated time periods.

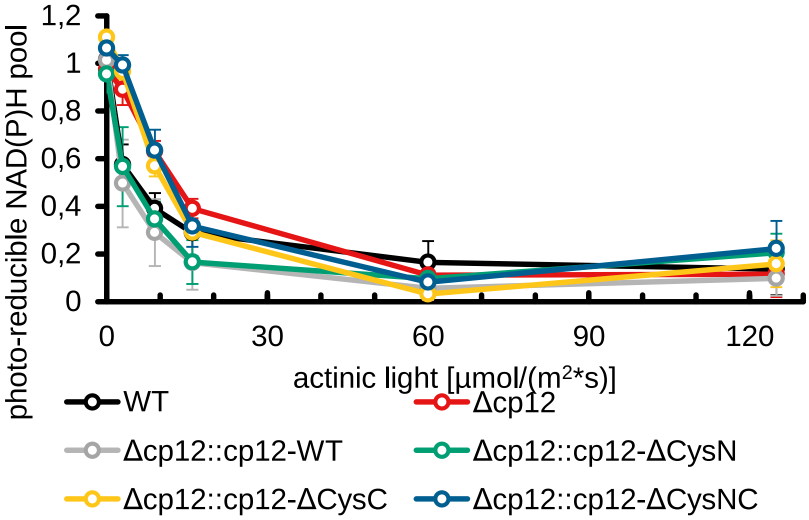

Supplementary Figure S10: Photo-reducible NAD(P)H level in different strains at increasing light conditions.

Cells of strains expressing different CP12 variants were acclimated to different actinic light conditions. Then, they were used for measurements in the NADPH-Modul (Walz, Germany) to determine the NAD(P)H reduction in darkness after a 600 ms long strong actinic light pulse.

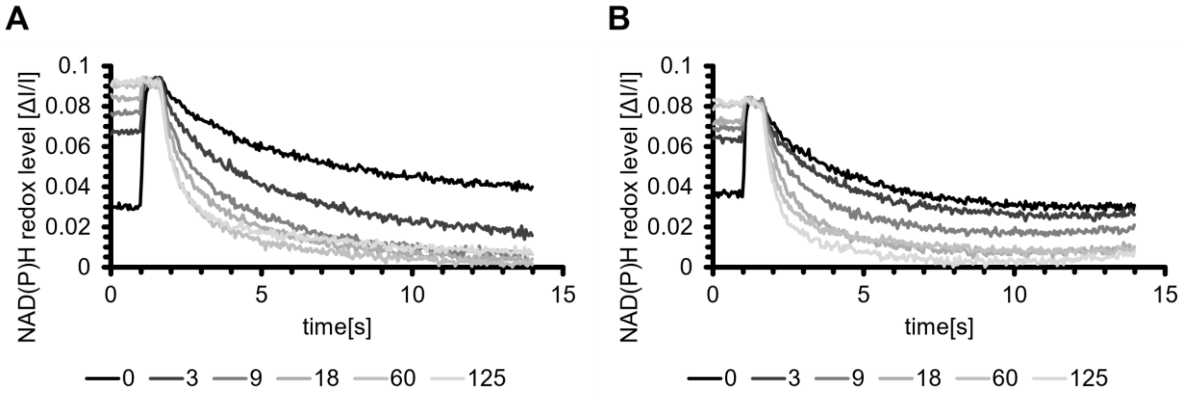

Supplementary Figure S11: NAD(P)H oxidation kinetics in GapDH-eYFP and PRK-eYFP cells adapted to different light intensities

NAD(P)H redox changes measured *in vivo* via NAD(P)H fluorescence in GapDH-eYFP (**A**) or PRK-eYFP cells (**B**). The NAD(P)H pool was reduced to its maximum with a strong actinic light pulse (600 ms) and its re-oxidation in darkness was observed. The measured cells were pre-acclimated to the 5 different actinic light intensities or to darkness.
